# Comprehensive Evaluation of Growth Performance, Hematological Parameters, Antioxidant Capacity, Innate Immunity, and Disease Resistance in Crucian Carp (*Carassius auratus*) Lacking Intermuscular Bones

**DOI:** 10.3390/antiox14040443

**Published:** 2025-04-08

**Authors:** Ran Zhao, Jianqiang Zhu, Shaowu Li, Zhipeng Sun, Tingting Zhang, Jing Wang, Xianhu Zheng, Youyi Kuang, Di Wang

**Affiliations:** Key Laboratory of Aquatic Animal Diseases and Immune Technology of Heilongjiang Province, Heilongjiang River Fisheries Research Institute, Chinese Academy of Fishery Sciences, Harbin 150070, China; zhaoran@hrfri.ac.cn (R.Z.); jqz9809@163.com (J.Z.); lishaowu@hrfri.ac.cn (S.L.); sunzhipeng@hrfri.ac.cn (Z.S.); zhangtingting@hrfri.ac.cn (T.Z.); wangjing@hrfri.ac.cn (J.W.); zhengxianhu@hrfri.ac.cn (X.Z.)

**Keywords:** *Carassius auratus*, a new strain lacking intermuscular bones, growth performance, iron overload, immune function

## Abstract

The presence of intermuscular bones severely affects the edibility and value-added processing of crucian carp (*Carassius auratus*), becoming a constraint to the high-quality development of its industry. Our previous study identified *bmp6* as the key osteogenic regulator and successfully developed a new crucian carp strain lacking intermuscular bones (WUCI) using CRISPR/Cas9 technology. To accelerate its industrialization, we comprehensively assessed WUCI’s growth performance, hematological parameters, antioxidant capacity, innate immunity, and disease resistance. The results demonstrated that the WUCI exhibited significant growth performance compared to the wild-type crucian carp (WT), with significantly higher weight gain (WG) and specific growth rate (SGR) (*p* < 0.05) from one month to four months of age. The α-amylase (α-AL) activity of the liver and intestines of WUCI was significantly higher than that of WT. WUCI also displayed enhanced intestinal antioxidant capacity, with superoxide dismutase (SOD) and catalase (CAT) activities significantly higher than those in WT (*p* < 0.05). The malondialdehyde (MDA) content in the spleen of WUCI was significantly lower than that of WT (*p* < 0.05); no differences were observed in the liver and intestines (*p* > 0.05). Additionally, hepatic acid phosphatase (ACP) activity in WUCI was significantly higher than that in WT (*p* < 0.05). In contrast, splenic ACP and intestinal alkaline phosphatase (ALP) activities were significantly lower than those in WT (*p* < 0.05). Notably, the iron concentration in the serum was significantly higher in WUCI than in the WT (*p* < 0.05). Meanwhile, WUCI exhibited significantly lower a expression of *hepcidin*, *TF*, and *TFR1* mRNA in the liver compared to WT (*p* < 0.05), while *FPN* mRNA expression was significantly higher (*p* < 0.05). Routine blood tests revealed significantly lower WBC in WUCI compared to that of WT (*p* < 0.05). Following an *Aeromonas hydrophila* challenge, WT demonstrated a rapid transcriptional induction of pro-inflammatory cytokines (*TNF-α*, *IL-1β*, *IL-6*) and immunoregulatory mediators (*IL-10*, *TGF-β*), with mRNA levels reaching maximal expression at 24 h post-infection (hpi) followed by progressive attenuation. In contrast, WUCI exhibited a delayed immune activation profile characterized by the peak expression of *TNF-α*, *IL-1β*, *IL-6*, and *IL-10* transcripts after 72 hpi, with the maximum transcript abundance remaining lower than corresponding peak values observed in WT at 24 hpi. Finally, we observed that the mortality rate of WUCI was slightly higher post *A. hydrophila* infection when compared to WT, but was not significant (*p* > 0.05). In conclusion, this study provides a comprehensive evaluation of WUCI, revealing its distinct growth advantages, physiological adaptations, and immune function, presenting its potential for aquaculture breeding applications.

## 1. Introduction

In recent years, CRISPR/Cas9 has emerged as a powerful tool for targeted genome editing and gene function studies, offering unprecedented assistance in accelerating genetic improvement of economic traits in aquaculture when combined with conventional selective breeding. To date, this technology has been widely applied in gene function and genetic breeding research for many aquaculture species, including *Cyprinus carpio* L. [1,2], *Danio rerio* [3,4,5], *Oryzias latipes* [6,7,8], *Ctenopharyngodon idellus* [9,10], enabling the development of desirable traits such as increased growth rate, controlled sex determination, altered skin color, and improved disease resistance.

Bone morphogenetic protein 6 (bmp6) serves as a key regulator in skeletal development, orchestrating both bone and cartilage formation, and osteogenic differentiation process [11]. During longitudinal bone growth, *bmp6* precisely modulates chondrocyte proliferation and maturation through conserved molecular mechanisms [12]. Lu et al. [13] studied miR-451a knockout mice and discovered that *bmp6* acts as a direct target of miR-451a-mediated negative regulation, which controls SMAD1/5/8 signaling activation during osteoblastogenesis, ultimately leading to an increase in bone mass. Studies in zebrafish revealed that the *bmp6* knockdown induces *sik1* up-regulation and activates TNF-α/NF-κB signaling cascades, impairing osteoblast development and intermuscular bones (IMB) formation [14]. Furthermore, in *bmp6*-deficient Silver Carp (*Hypophthalmichthys molitrix*), dysregulated expression of osteoclastogenic markers (*tnfα*, *fos*, *ctgf*) and osteoblastic regulators (*tgfb2*, *tgfbr1*) could lead to rib abnormalities [15].

Crucian carp (*Carassius auratus*), a commercially vital freshwater species in Chinese aquaculture, is prized for its rapid growth rate, abbreviated reproductive cycle, and high nutritional value [16]. Our previous study successfully obtained a new strain (WUCI) without IMB by knocking out two *bmp6* orthologs using CRISPR/Cas9 technology, and confirmed this trait in F_3_ progeny was stable and inheritable [17]. We found that WUCI exhibited no significant differences in reproductive performance or meat quality compared to WT. In addition, muscle tissue analysis revealed significant enrichment of metabolites associated with thiamine metabolism, nicotinate, and nicotinamide metabolic pathways in WUCI specimens, which play crucial roles in anti-aging processes, antioxidant defense mechanisms, and radiation damage mitigation, suggesting potential health-promoting benefits for human consumers. Despite these advancements, critical aquaculture-relevant parameters including physiological performance and immunological competence in this new strain remain uncharacterized.

To systematically evaluate the biosafety profile of WUCI, we conducted a comprehensive comparative analysis encompassing growth metrics, hematological parameters, digestive physiology, antioxidant defense systems, innate immune functions, and pathogen resistance against *Aeromonas hydrophila* between WUCI and wild-type crucian carp (WT). This multidimensional assessment aims to establish an integrated understanding of WUCI’s biological performance while providing critical data for its risk evaluation in aquaculture applications.

## 2. Materials and Methods

### 2.1. Experimental Animals

The WUCI and WT carp used in this study were maintained under standardized aquaculture conditions at the Hulan Experimental Station of HRFRI. After hatching, larvae were fed *Artemia nauplii* 4 times daily until reaching a body length of 2–3 cm, after which, they were transferred to 500 m^2^ ponds and fed artificial feed 3 times daily. Both groups were cultured under identical conditions: water temperature of 24 ± 2 °C, pH of 8.0 ± 0.5, and dissolved oxygen levels of 6.0–10 mg/L.

### 2.2. Growth Performance Evaluation and Sample Collection

A total of 600 one-month-old WUCI (initial average body weight of 4.6 ± 0.42 g) and 600 one-month-old WT (initial average body weight of 4.2 ± 0.55 g) were randomly selected for this study. WUCI and WT were each divided into three groups and distributed across six aquaculture cages of identical specifications, with each cage containing 200 fish. All six cages were placed in the same pond to ensure a consistent rearing environment. At 4 months of age, fish were fasted for 24 h prior to sampling, and 30 fish per cage were randomly selected for the determination of BW and standard length (SL), as well as the calculation of weight gain (WG), specific growth rate (SGR), condition factor (CF), and final body mass index (BMI). A total of 15 fish from each group were then anesthetized, and blood was collected from the tail vein using 2.5 mL syringes with heparin sodium anticoagulation, while the liver, intestine, and spleen tissues were aseptically excised, placed into RNases-free tubes, and stored at −80 °C.

### 2.3. Blood Sample Processing and Analysis

The collected blood was divided into two aliquots. The first aliquot was analyzed using the animal blood cell analyzer (BC-2800Vet, Mindray, Shenzhen, China) to evaluate parameters including white blood cell count (WBC), red blood cell count (RBC), hemoglobin (HGB), hematocrit (HCT), and others. The second aliquot was allowed to settle for 2 h at 4 °C, then centrifuged at 2500 rpm for 10 min to collect the supernatant. The serum was analyzed using the biochemical analyzer (BS-240Vet, Mindray) along with various test kits produced by Mindray Biomedical Electronics Co., including kits for measuring aspartate aminotransferase (AST), alkaline phosphatase (ALP), high-density lipoprotein cholesterol (HDL-C), low-density lipoprotein cholesterol (LDL-C), total cholesterol (TC) and triglyceride (TG), glucose (Glu), iron (Fe), and calcium (Ca). Moreover, all samples from each group were analyzed in triplicate (N = 15 per group).

### 2.4. Detection of Digestive, Antioxidant, and Immune-Related Enzymes

The liver, intestines, and spleen from 5 fish per cage were sampled, and each kind of tissue was mixed together and regarded as one sample. Finally, a total of 15 fish from triplicate cages of each group were sampled, and 3 samples of each kind of tissue were used for experimental replication. The samples, homogenized in an ice-cold phosphate buffer (1:10, *w*/*v*), were centrifuged at 3000× *g* for 10 min at 4 °C. The supernatants were collected to assay digestive enzymes, nonspecific immunity markers, and antioxidant enzymes. The activities of trypsin, α-amylase (α-AL), lipase (LPS), superoxide dismutase (SOD), catalase (CAT), acid phosphatase (ACP), and alkaline phosphatase (AKP), as well as the content of malondialdehyde (MDA), were measured according to the manufacturer’s protocols (Suzhou Grace Biotechnology Co., Suzhou, China).

### 2.5. Expression of Iron Metabolism and Immune-Related Genes

The total RNA was extracted from the liver and spleen tissues using Trizol reagents (Invitrogen, Waltham, MA, USA), with quality verified by 1% agarose gel electrophoresis and purity assessed via spectrophotometry (Nanodrop 8000, Thermo Fisher, Waltham, MA, USA), respectively. cDNA synthesis was performed with the PrimerScript^TM^ RT reagent Kit (Takara, Shiga, Japan). qRT-PCR was conducted in a quantitative thermal cycler, and the melting curve analysis confirmed amplification specificity. β-actin served as the reference gene due to its stable expression levels across experimental conditions. The primer sequences are listed in Table 1, and relative expression levels were calculated using the 2^−ΔΔCt^ method [18].

### 2.6. Aeromonas Hydrophila Challenge Test

Prior to the experiment, *A. hydrophila* stored at −80 °C was inoculated onto TSA plates and cultured at 28 °C for 24 h. Subsequently, a single colony was picked and inoculated into TSB medium, followed by shaking at 120 rpm for 18 h until reaching the logarithmic growth phase. Afterwards, *A. hydrophila* were harvested by centrifugation at 4000 rpm for 10 min, then washed and resuspended in sterile saline to prepare a bacterial suspension with a final concentration of 5 × 10^6^ CFU/mL. This challenge concentration was optimized through preliminary experiments to ensure effective infection while maintaining sufficient host survival for subsequent sampling.

A total of 150 fish from each group used for *A. hydrophila* challenging were taken from the ponds to the laboratory and temporarily cultured for 7 days to stabilize. A total of 90 fish from triplicate cages of WUCI and WT were randomly selected to be injected with *A. hydrophila* (5 × 10^6^ CFU/mL, 125 μL per fish), with 30 fish from each group selected to observe the mortality rate, and 60 fish from each group retained for sampling to conduct subsequent experimental analysis. The mortality rate of the two groups was recorded during this period and samples were collected on the 1st, 3rd, and 7th day for gene expression determination. Nine fish from triplicate cages per group were sampled at each time point and each kind of tissue was mixed into three samples for experimental replication. The RNA extraction, cDNA synthesis, qPCR conditions, and result calculation methods were performed as previously described.

### 2.7. Statistical Analysis

Statistical analysis was conducted in SPSS 22.0. All data were presented as mean ± SD. When comparing two independent groups, an independent samples *t*-test was used, whereas for three or more groups, a one-way analysis of variance (ANOVA) followed by Duncan’s multiple comparison test was applied to determine statistical significance (*p* < 0.05) between groups.

## 3. Results

### 3.1. Growth Performance

The growth performance of WUCI is presented in Table 2. Compared with WT, WUCI exhibited significantly higher final BW and SL, WG, and SGR (*p* < 0.05). Furthermore, no significant differences were observed in final CF and BMI between the two groups (*p* > 0.05).

### 3.2. Blood Parameters

Routine blood tests were conducted on WUCI and WT and revealed significantly lower WBC in WUCI compared to that of WT (*p* < 0.05), with other cellular parameters remaining comparable (Table 3).

Serum biochemical analysis (Table 4) showed elevated lipid levels in WUCI, including HDL-C, LDL-C, TC, and TG (*p* < 0.05). Conversely, Glu levels in WUCI were significantly lower than those of WT (*p* < 0.05). Notably, the serum iron concentration was significantly higher in WUCI than in WT (*p* < 0.05).

### 3.3. Digestive, Antioxidant Capacity, and Immune Functions

**Figure 1:**
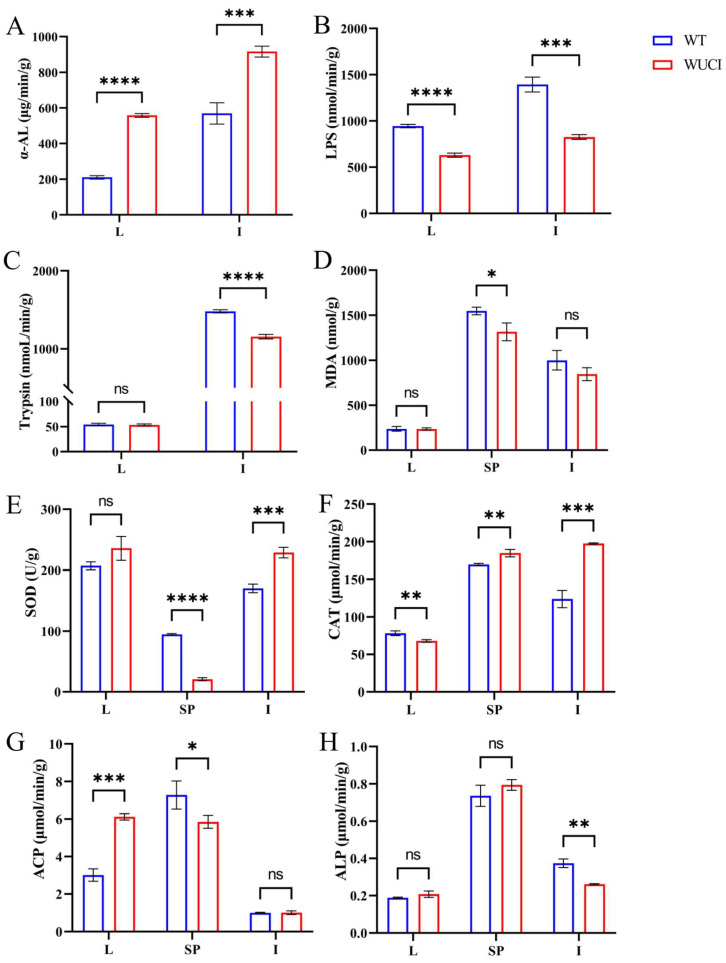
The results of digestive, antioxidant capacity, and immune functions in the liver (L), intestines (I), and spleen (SP) of WT and WUCI. (**A**) The activities of α-AL. (**B**) The activities of LPS. (**C**) The activities of Trypsin. (**D**) The contents of MDA. (**E**) The activities of SOD. (**F**) The activities of CAT. (**G**) The activities of ACP. (**H**) The activities of ALP. Data are presented as mean ± SD (*n* = 3). WT: the wild-type crucian carp; WUCI: the new strain without intermuscular bones; *: *p* < 0.05; **: *p* < 0.01; ***: *p* < 0.001; ****: *p* < 0.0001; ns: no significance.

Comparative analysis of digestive capacity between WUCI and WT revealed that the α-AL activity of the liver and intestines of WUCI was significantly higher than that of WT (Figure 1A). In contrast, lipase (LPS) activity demonstrated inverse patterns across the two tissues (Figure 1B). Furthermore, the trypsin activity of the intestines of WUCI was significantly lower than that of WT, while no significant difference between the liver of the two groups was observed (Figure 1C).

The results of evaluating antioxidative indices of WUCI are presented in Figure 1D–F. The MDA content in the spleen of WUCI was significantly lower than that of WT (*p* < 0.05), while the intestinal MDA was also lower than that of WT, but there was no significant difference (*p* > 0.05). The SOD activities in the intestines and CAT activities in both the spleen and intestines of WUCI were significantly higher than those observed in WT. In contrast, the activities of SOD in the spleen and the activities of CAT in the liver of WUCI were significantly lower than those of WT.

Nonspecific immune function analysis revealed that ACP activity in the WUCI’s liver was significantly higher than in WT (*p* < 0.001), while the activity of ACP in the spleen was significantly lower than that of WT (*p* < 0.05). Compared to WT, the ALP activity in the intestines of WUCI was significantly lower (*p* < 0.01). Moreover, the activity of ALP showed no significant difference in the liver and spleen between WUCI and WT (*p* > 0.05).

### 3.4. Iron Metabolism and Immune-Related Gene Expression

**Figure 2:**
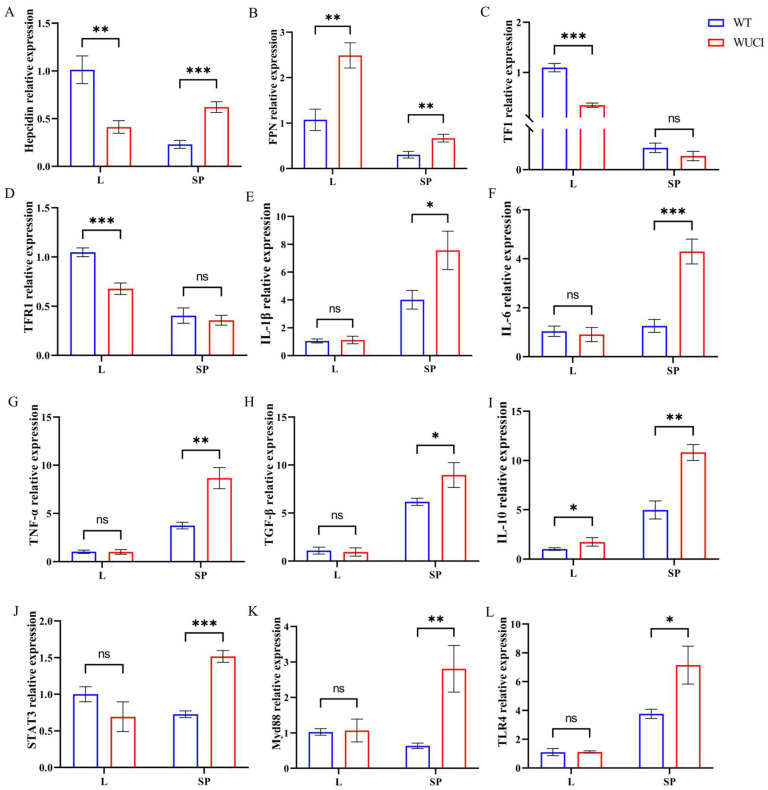
The relative mRNA expression of iron metabolism and immune-related genes in the liver (L) and spleen (SP) of WT and WUCI. (**A**) *Hepcidin*. (**B**) *FPN*. (**C**) *TF*. (**D**) *TFR1*. (**E**) *IL-1β*. (**F**) *IL-6*. (**G**) *TNF-α*. (**H**) *TGF-β*. (**I**) *IL-10*. (**J**) *STAT3*. (**K**) *Myd88*. (**L**) *TLR4*. Data are presented as mean ± SD (*n* = 3). WT: the wild-type crucian carp; WUCI: the new strain without intermuscular bones; *: *p* < 0.05; **: *p* < 0.01; ***: *p* < 0.001; ns: no significance.

As shown in Figure 2, the mRNA expression level of iron metabolism-related genes of WUCI was highly affected by the knockout of bmp6, with tissue-specific differences between the liver and spleen. In the liver, WUCI exhibited significantly lower expression of hepcidin, TF, and TFR1 mRNA compared to WT (*p* < 0.05), while FPN mRNA expression was significantly higher (*p* < 0.05). In contrast to the liver, the expression levels of hepcidin and FPN mRNA in the spleen of WUCI were significantly higher than that of WT (*p* < 0.05). Furthermore, analysis of immune-related gene expression revealed tissue-specific differences between WUCI and WT. In the spleen, WUCI displayed significantly higher mRNA expression levels of multiple immune-related genes (*p* < 0.05), including *IL-1β*, *IL-6*, *TNF-α*, *TGF-β*, *STAT3*, *IL-10*, *Myd88*, and *TLR4*. However, the liver showed a more limited immune response, with only *IL-10* mRNA expression being significantly higher in WUCI compared to WT (*p* < 0.05).

### 3.5. Survival Rate

**Figure 3:**
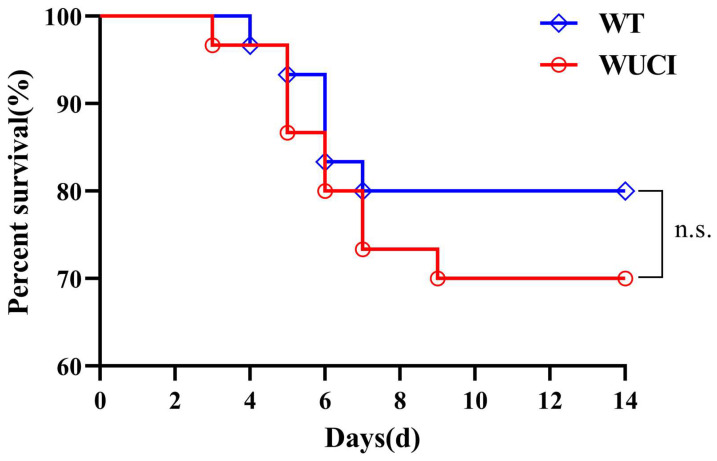
The results of survival of WUCI and WT after challenge with *A. hydrophila* which lasted for 14 days. n.s.: no significance. *n* = 30.

The survival rates of WUCI and WT infected with *A. hydrophila* are shown in Figure 3. The survival rates of WUCI were lower than those of WT, but there were no significant difference (*p* > 0.05).

### 3.6. Immune-Related Gene Expression After Injection of A. hydrophila

**Figure 4:**
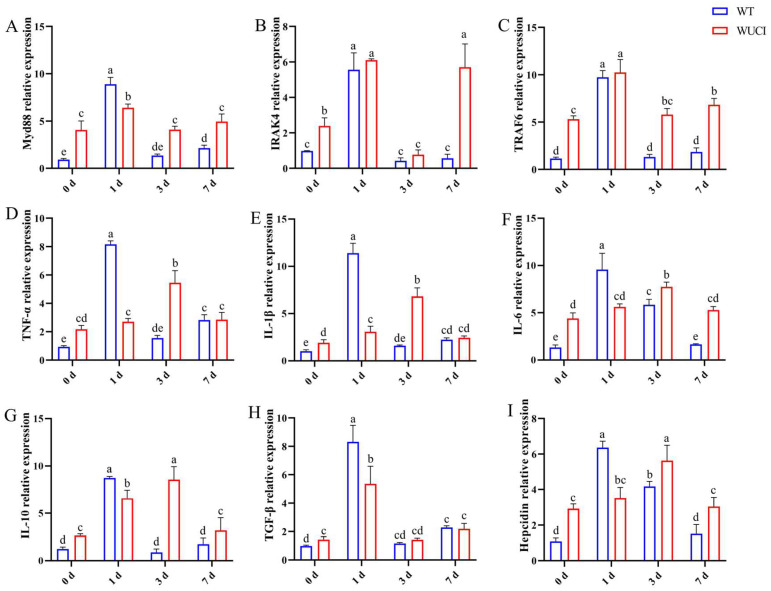
The relative mRNA expression of immune-related genes in the spleen of WT and WUCI post A. hydrophila injection. (**A**) *Myd88*. (**B**) *IRAK4*. (**C**) *TRAF6*. (**D**) *TNF-α*. (**E**) *IL-1β*. (**F**) *IL-6*. (**G**) *IL-10*. (**H**) *TGF-β*. (**I**) *Hepcidin*. Data are presented as mean ± SD (*n* = 3). WT: the wild-type crucian carp; WUCI: the new strain without intermuscular bones; different letters indicate a significant difference (*p* < 0.05).

The immune-related gene mRNA expression in the spleen of WT and WUCI after injection of *A. hydrophila* is shown in Figure 4. The mRNA expression patterns of these genes are highly similar over time. The expression of *Myd88*, *IRAK4*, and *TRAF6* mRNA in both WT and WUCI significantly increased and reached their peak when they were infected with *A. hydrophila* on day 1 post-infection. However, the peak mRNA expression level of *Myd88* in WUCI was significantly lower than that in WT. Furthermore, the expression of *IRAK4* and *TRAF6* mRNA significantly increased again in WUCI on day 7. The expression of *TNF-α*, *IL-1β*, *IL-6*, *IL-10*, *TGF-β*, and *hepcidin* mRNA in WT increased sharply and reached a peak on day 1 post-infection with *A. hydrophila*, followed by a decrease. In contrast, the expression of *TNF-α*, *IL-1β*, *IL-6*, *IL-10*, and *hepcidin* mRNA did not peak until 3 days post-infection in WUCI, and even at their peak on day 3, the mRNA expression levels of WUCI were generally lower than the peak levels observed in WT on day 1.

## 4. Discussion

### 4.1. Enhanced Growth, Altered Metabolism, and Immune Adaptations

In the present study, WUCI with significantly higher WG and SGR exhibited improved growth performance compared to WT over a time period of one to four months. The biochemical parameters in fish serum could mirror their nutritional condition and are commonly utilized to assess their metabolic and physiological states [19,20]. TC and TG levels in serum are essential lipid metabolism parameters in fish. Bone morphogenetic protein (BMP) has been identified as a new member of the TGF-β superfamily and has been found to be associated with fat differentiation and energy balance [21]. In rat H4IIE hepatoma cells, BMP6 inhibits gluconeogenesis and glucose output by down-regulating PepCK expression; meanwhile, the study found that mice treated with recombinant BMP6 exhibited a decrease in plasma TG and TC within 6 days [22]. It has been demonstrated in mouse models that exogenous BMP4 promotes lipid turnover by up-regulating genes involved in lipid metabolism, thereby reducing serum TG and body weight [23]. In addition, studies have found that *bmp8a* knockout in zebrafish leads to the accumulation of liver TG by down-regulating the phosphorylation of AMPK and acetyl CoA carboxylase (ACC), resulting in higher levels of blood TG and TC compared to WT zebrafish [24]. In this study, WUCI had significantly higher levels of HDL-C, LDL-C, TC, and TG in serum, and showed lower lipase activity in the liver and intestines compared to WT. Hepatic lipase hydrolyzes PS and TG of plasma lipoproteins, regulating of plasma TG levels [25,26]. In this study, WUCI, due to the absence of Bmp6, reduced the activity of the key enzyme in lipid metabolism, lipase, weakened its ability to hydrolyze TG, and led to an increase in serum TG levels. Amylase is one of the most important digestive enzymes, regulating carbohydrate metabolism [27]. Moreover, α-amylase is regarded as a key factor affecting the growth of many aquatic animals [28]. WUCI showed higher α-AL in the liver and intestines compared to WT, indicating that WUCI has enhanced carbohydrate digestibility, which may contribute to the improved growth performance of WUCI.

In normal creatures, the production and elimination of reactive oxygen species (ROS) sustains a state of dynamic equilibrium. Nevertheless, an overabundance of ROS can damage DNA and engage in specific reactions with lipids, inducing the generation of MDA [29,30]. The degree of oxidative stress within the body could be assessed through the measurement of MDA, which serves as a lipid peroxidation indicator [31,32]. A marker of oxidative damage, MDA, only differed in spleen, suggesting no significant increase in oxidative damage due to the loss of bmp6. As the foremost line of defense against ROS, SOD is rapidly activated when the body is exposed to oxidative stress, eliminating superoxide free radicals [33,34]. CAT is regarded as the key enzyme for neutralizing ROS within the biological antioxidant defense system and safeguards cells against oxidative damage [35,36]. The SOD and CAT activities in the intestines of WUCI were significantly higher than those of WT in this study, indicating that the antioxidant capacity of the intestine of WUCI was stronger. However, the spleen of WUCI showed a compensatory mechanism in terms of antioxidant activity, with significantly higher CAT activity and significantly lower SOD activity compared to those of WT. In addition, the CAT activity in the liver of WUCI was significantly lower than that of WT, suggesting that the antioxidant capacity of the liver of WUCI may be inferior to that of WT.

White blood cells (WBC) are responsible for resisting infectious pathogens caused by microbes [37]. Previous studies have reported that dietary supplementations with astaxanthin and Ginkgo biloba leaf extract show enhanced WBC levels in common carp, high resistance to A. hydrophila, and reduced mortality from infection [37,38]. Notably, WUCI in this study displayed significantly lower WBC than that of WT, suggesting that WUCI may have weak resistance to pathogens. ACP and ALP play important roles in the immune system, participating in the first line of nonspecific immunity in aquatic animals [39,40,41]. Interestingly, WUCI exhibited tissue-specific variations in nonspecific immune enzyme activities compared to WT, with higher ACP activity in the liver but lower ACP activity in the spleen, and lower ALP activity in the intestines. We speculate that WUCI has developed unique antioxidant and immune adaptation mechanisms diverging from WT.

### 4.2. Dysregulated Iron Homeostasis by bmp6-Knockout

In vertebrates, the homeostasis of plasma and extracellular iron levels, along with overall body iron levels, is sustained through the interplay between hepcidin and cellular iron exporter ferroportin (FPN) [42]. FPN is the only known iron exporter, playing a crucial role in exporting iron into the blood from duodenal enterocytes involved in dietary iron uptake, splenic and hepatic macrophages that recycle iron, and hepatocytes responsible for iron storage [43]. WUCI exhibited a high expression of FPN mRNA compared to WT, which exhibited that the body accelerated the process of iron efflux from hepatocytes to blood. Transferrin (TF) is mainly synthesized in the liver and released to the blood [44]. Under normal conditions, most of the iron in the plasma is bound to TF, and iron–TF complexes enter cells via a transferrin receptor (TFR)-mediated endocytic pathway, completing cellular iron uptake [45]. Due to TFR1 acting as a receptor for iron incorporation into cells, its expression is regulated by cellular iron status. The expression of TFR1 is reduced in conditions of cellular iron excess [46]. Our previous study found that the iron content in the liver of WUCI due to bmp6 knockout was also higher than that in WT [17]. Excessive production of ROS mediated by iron overload can cause oxidative damage to biomolecules [47]. WUCI exhibited significant lower expression of TF and TFR1 mRNA in the liver compared with WT, indicating that due to the high iron content in the liver of WUCI, the organism inhibited hepatocytes from taking up iron from the blood to prevent liver damage.

Unfortunately, our study found that the expression of hepcidin mRNA in the liver of WUCI was lower than that of WT. The binding of hepcidin to the cellular iron exporter FPN leads to its internalization and degradation, preventing the release of iron into the plasma [48]. The BMP–SMAD pathway regulates the transcription of hepcidin, controlling its expression in reaction to iron [49]. Tissue iron loading triggers an increase in bmp6 generation in endothelial cells of liver [50], which activates SMAD1/5/8 and SMAD4, and then initiates hepcidin transcription [51]. In our study, due to the knockout of bmp6 in WUCI, the expression of hepcidin mRNA decreased; thus, hepcidin failed to effectively bind to FPN and terminate the process of iron release into the blood. This explains why the iron content in the serum of WUCI was significantly higher than that of WT. Research with bmp6-null mice revealed that these mice had reduced hepatic hepcidin expression and spleen iron content, and increased serum and liver iron content [52]. Similarly, FPN expression was markedly enhanced in the livers and spleens of bmp6−/− mice compared with those of wild-type controls [53], which was consistent with our research findings. Although serum iron levels in WUCI were elevated and may indicate iron overload, MDA content was lower in different tissues. We speculated that severe oxidative damage may not have occurred, and the iron overload was within the body’s tolerance range.

Interestingly, despite lacking bmp6, these mice maintained the ability to induce the hepcidin response to inflammation. They increased hepcidin expression as a reaction to inflammatory cytokines, indicating that the response pathways induced by iron and cytokines to hepcidin were initially distinct [53,54]. Previous studies have confirmed that IL-6 [55,56] and IL-1 [57,58] stimulate hepcidin transcription through STAT3 signaling during infection and inflammation [59,60,61]. We speculate that the high expression of hepcidin mRNA in the spleen of WUCI may be achieved through the inflammatory response pathway, which is different from the liver.

### 4.3. Weakened A. hydrophila Resistance

*A. hydrophila* infections cause visible damage to the exterior of fish, leading to severe internal infections, and reducing the immune capability of fish [62]. In this study, we found that the mortality rate of WUCI was slightly higher than that of WT infected with A. hydrophila lasting 14 days. Immune-related genes play essential roles in regulating the immune system. Generally, microbial components of invading bacteria can be recognized by toll-like receptors (TLRs) [63], which then initiate intracellular signaling by activating Myd88 [64]. Myd88 serves as its main adaptor molecule, getting involved in the regulation of TLR signaling by recruiting IRAK4 [65,66]. Activated IRAK4 recruits downstream, signaling the molecule TRAF6, involved in inducing proinflammatory cytokines through NF-κB pathway activation [67]. Following A. hydrophila infection in the spleen of blunt snout bream, the expression of Myd88 and TRAF6 showed initial up-regulation, and subsequently normalized in the later stage [68]. In Qihe crucian carp (Carassius gibelio), Myd88 and TRAF6 mRNA levels peaked 24 h after A. hydrophila infection [69], which was matched with our findings. Furthermore, the expression of TLR4, IL-1β, IL-6, and IL-10 was up-regulated at 24 h post infection in the common carp (Cyprinus carpio) spleen in response to A. hydrophila [70]. The TLR4/Myd88/NF-κB pathway plays a vital role in regulating inflammation [71]. Immune cells secrete both pro-inflammatory and anti-inflammatory cytokines to regulate immune responses, repair damaged tissues, and defend against infection [72,73]. As key pro-inflammatory cytokines, TNF-α and IL-1β mediate inflammatory responses while playing a crucial role in host defense against microbial pathogens, including both bacterial and viral agents. [74,75]. The occurrence of an inflammatory response can increase the expression level of pro-inflammatory cytokines, including TNF-α and IL-1β, whereas high expression of anti-inflammatory cytokines, including IL-10 and TGF-β, can relieve inflammation by inhibiting the release of inflammatory cytokines [76,77]. Previous transcriptome results revealed that the expression of IL-1β, TNF-α, and IL-10 in the spleen of grass carp (Ctenopharyngodon idellus) infected with A. hydrophila was up-regulated in the early stages [78]. In this study, WUCI exhibited a delayed inflammatory response to A. hydrophila infection compared to WT, and the peak levels of TNF-α, IL-1 β, IL-10, and TGF-β mRNA were lower than those of WT, indicating that although WUCI had a certain degree of resistance to A. hydrophila infection, its overall immune response was inferior to that of WT, making it more susceptible to infection or more difficult to control infection effectively.

## 5. Conclusions

This study reveals several significant findings regarding WUCI compared to WT. WUCI demonstrated improved growth performance, along with distinct metabolic characteristics, including elevated serum lipid levels and enhanced carbohydrate digestibility. Furthermore, *bmp6* knockout in WUCI disrupts hepatic hepcidin regulation, leading to a significant increase in serum iron content, while compensatory inflammatory pathways in the spleen maintain hepcidin expression, preventing severe oxidative damage within physiological tolerance. However, WUCI showed lower WBC levels and an increased susceptibility to *A. hydrophila* infection, with delayed and weaker inflammatory responses compared to WT. In general, while WUCI exhibited improved growth and unique metabolic characteristics, its disease resistance was slightly weaker to WT, but there was no significant difference in infection mortality rate compared to WT. These findings highlight the multifaceted nature of gene editing, where targeted trait enhancement may inadvertently affect other physiological systems through complex regulatory networks. The research contributes to our understanding of the complex mechanisms of genetic regulation in fish, providing valuable insights for future genetic modification strategies and potential applications in aquaculture and fish breeding.

### Acknowledge Limitations

In enzyme activity and gene expression assays, we used three to five fish as a sample, with three samples collected from three replicate cages in the WUCI and WT groups. This analytical method provides more power to detect treatment effects, but it may increase the risk of Type I errors and fails to effectively account for biological variation.

## Figures and Tables

**Table 1:** Primer sequences of unigenes verified by qRT-PCR.

Gene Name	Accession No.	Primer Type	Nucleotide Sequences (5′-3′)
*18S*	FJ710820.1	F	CCGACCCTCCCTCACG
		R	GCCTGCTGCCTTCCTTG
*TLR4*	KT966378.1	F	TCACTCTTGGTTTACAGGTTTCAG
		R	GTTGGAGGCGATGGACTT
*Myd88*	KF767100.1	F	TGAGGCGATTCCAGTAACAGC
		R	TTGCCTCTGGACGAGTTTCC
*IRAK4*	XM_026202045.1	F	GCGTCCTGCTGCCTGAT
		R	CCTCTGAACACGATGCCAA
*TRAF6*	KF767099.1	F	AGACCAGCAAGGCTATGACG
		R	GCCGAGCGAAGACCCA
*TNF-α*	EU069818.1	F	CATTCCTACGGATGGCATTTACTT
		R	CCTCAGGAATGTCAGTCTTGCAT
*IL1β*	AJ249137.1	F	GATGCGCTGCTCAGCTTCT
		R	AGTGGGTGCTACATTAACCATACG
*IL10*	HQ259106.1	F	GCTTCTACTTGGACACCATTCT
		R	TCTTTATGCTGGCGAACTCA
*IL 6*	DQ861993.1	F	AAGCCAGTCAGGGAGATTTT
		R	GGGTTGGTTGGAGGATTTAA
*TGF-β*	EU086521.1	F	GTACACTACGGCGGAGGATTG
		R	CGCTTCGATTCGCTTTCTCT
*Hepcidin*	XM_026227843.1	F	AGATCACAGCCGTTCCCTT
		R	GCTTTGACGCTTCACCCT
*FPN*	XM_026217613.1	F	TCGACCAGCTAACCAACATT
		R	CCAACCCGAGATAAAACCA
*TFR1*	XM_026206355.1	F	TGGCTTGCCCAGTATTCC
		R	GCCGTCATCACCGAGTTT
*STAT3*	XM_026202757.1	F	CAGCCTGTCAGCAGAGTTCA
		R	TCAGGTGCAGTTCTTCGGTC
*TF*	XM_026283020.1	F	CTCTTCTGCTGCTTTTGGTG
		R	GCATGGCCTCATAATAATCTTC

**Table 2:** Growth parameters of WT and WUCI.

Biometric Parameters	WT	WUCI
IBW (g)	4.2 ± 0.55	4.6 ± 0.42
FBW (g)	56.11 ± 4.31 ^b^	74.23 ± 9.91 ^a^
FSL (cm)	14.87 ± 0.40 ^b^	16.50 ± 0.73 ^a^
WG (%)	1235.95 ± 202.91 ^b^	1513.70 ± 261.05 ^a^
SGR (%/day)	2.88 ± 0.17 ^b^	3.09 ± 0.18 ^a^
FCF	1.71 ± 0.19	1.65 ± 0.31
FBMI (kg/m^2^)	2.54 ± 0.24	2.73 ± 0.44

Note: Different line letters indicate significant difference (*p* < 0.05). IBW, initial body weight; FBW, final body weight; FSL, final standard length; WT, the wild-type crucian carp; WUCI, the new strain without intermuscular bones. Weight gain (WG, %) = 100 × (FBW − IBW)/IBW; specific growth rate (SGR, %/day) = 100 × [Ln(FBW) − Ln(IBW)]/days; FCF = 100 × FBW/FSL^3^ (FBW in g, FSL in cm); FBMI = FBW/FSL^2^. Data are presented as mean ± SD (*n* = 90).

**Table 3:** Hematological biomarkers.

Hematological Parameters	WT	WUCI
WBC (×10^9^/L)	211.98 ± 11.26 ^a^	193.5 ± 7.88 ^b^
RBC (×10^4^/μL)	54.67 ± 5.69	55.8 ± 6.72
HGB (g/L)	82.67 ± 2.08	87.67 ± 8.26
HCT (%)	10.27 ± 0.49	11.13 ± 0.55
MCV (fL)	192.15 ± 6.02	190.84 ± 4.83
MCH (pg)	168.3 ± 2.40	151.44 ± 10.80
MCHC (g/L)	881.5 ± 7.78	789.2 ± 43.14
RDW (%)	6.88 ± 0.37	7.74 ± 0.94
MPV (fL)	6.2 ± 0.39	5.97 ± 0.31
PDW (fL)	19.4 ± 0.64	18.87 ± 0.75
PCT (%)	0.02 ± 0.01	0.02 ± 0.01

Note: Different line letters indicate significant difference (*p* < 0.05). Data are presented as mean ± SD (*n* = 15).

**Table 4:** Biochemical biomarkers.

Hematological Parameters	WT	WUCI
AST (U/L)	106.29 ± 37.74	118.51 ± 42.01
ALP (U/L)	22.61 ± 6.26	23.53 ± 11.67
UREA (mmol/L)	0.47 ± 0.08	0.54 ± 0.12
LDL-C (mmol/L)	0.32 ± 0.07 ^b^	0.77 ± 0.36 ^a^
HDL-C (mmol/L)	1.33 ± 0.32 ^b^	2.20 ± 0.42 ^a^
TC (mmol/L)	2.04 ± 0.46 ^b^	4.44 ± 1.13 ^a^
TPⅡ (g/L)	18.09 ± 2.13	21.79 ± 6.31
TG (mmol/L)	0.98 ± 0.37 ^b^	1.46 ± 0.58 ^a^
CHE (U/L)	103.38 ± 25.29	91.33 ± 22.02
Glu (mmol/L)	25.85 ± 7.00 ^a^	9.65 ± 2.22 ^b^
Ca (mmol/L)	2.46 ± 0.22	2.35 ± 0.45
Fe (μmol/L)	7.11 ± 3.89 ^b^	26.60 ± 3.78 ^a^

Note: Different line letters indicate significant difference (*p* < 0.05). Data are presented as mean ± SD (*n* = 15).

## Data Availability

Data is contained within the article.
